# Multiple visual objects are represented differently in the human brain and convolutional neural networks

**DOI:** 10.1038/s41598-023-36029-z

**Published:** 2023-06-05

**Authors:** Viola Mocz, Su Keun Jeong, Marvin Chun, Yaoda Xu

**Affiliations:** 1 Visual Cognitive Neuroscience Lab, Department of Psychology, Yale University, 2 Hillhouse Ave, New Haven, CT 06520 USA; 2 Department of Neuroscience, Yale School of Medicine, New Haven, CT 06520 USA; 3 Department of Psychology, Chungbuk National University, Cheongju, South Korea

**Keywords:** Visual system, Object vision, Cognitive neuroscience, Perception, Neuroscience, Computational neuroscience, Network models

## Abstract

Objects in the real world usually appear with other objects. To form object representations independent of whether or not other objects are encoded concurrently, in the primate brain, responses to an object pair are well approximated by the average responses to each constituent object shown alone. This is found at the single unit level in the slope of response amplitudes of macaque IT neurons to paired and single objects, and at the population level in fMRI voxel response patterns in human ventral object processing regions (e.g., LO). Here, we compare how the human brain and convolutional neural networks (CNNs) represent paired objects. In human LO, we show that averaging exists in both single fMRI voxels and voxel population responses. However, in the higher layers of five CNNs pretrained for object classification varying in architecture, depth and recurrent processing, slope distribution across units and, consequently, averaging at the population level both deviated significantly from the brain data. Object representations thus interact with each other in CNNs when objects are shown together and differ from when objects are shown individually. Such distortions could significantly limit CNNs’ ability to generalize object representations formed in different contexts.

## Introduction

In everyday visual perception, objects are rarely encoded in isolation, but often with other objects appearing in the same scene. It is thus critical for primate vision to recover the identity of an object regardless of whether or not other objects are encoded concurrently. Both monkey neurophysiological and human fMRI studies have reported the existence of averaging for representing multiple objects at high-level vision. Specifically, in macaque inferotemporal (IT) cortex that is not category selective, neuronal response amplitude to a pair of unrelated objects can be approximated by the average response amplitude of each object shown alone^1^; similarly, in human occipital-temporal cortex, fMRI response pattern to a pair of unrelated objects can be predicted by the average fMRI response pattern of each object shown alone^2–7^. The whole is thus equal to the average of parts at high-level primate vision (note that responses in category-selective regions may exhibit both the averaging and winner-take-all responses depending on the stimuli shown, see^5,6,8^). Such a representational scheme can effectively avoid response saturation, especially for neurons responding vigorously to each constituent object, and prevent the loss of identity information when objects are encoded together^3^. This kind of tolerance to the encoding context, together with the ability of the primate high-level vision to extract object identity across changes in other non-identity features (such as viewpoint, position and size), has been argued as one of the hallmarks of primate high-level vision that allows us to rapidly recognize an object under different viewing conditions^9–11^.

In monkey neurophysiological studies, averaging was assessed at the single unit level by documenting the slope of single neuron response amplitudes to an object pair and the constituent objects. In fMRI studies, however, averaging was measured at the population level by correlating the voxel response patterns, and so it is not known whether averaging occurs at the individual voxel level. While averaging at the pattern level must stem from averaging at the individual voxel level, in a previous study, the slope of fMRI voxels to single and paired objects appeared to be substantially less than those found in macaque IT neurons. MacEvoy and Epstein^3^ presented single or paired objects to human observers. Rather than calculating the slope of each fMRI voxel in the human lateral occipital cortex (LO, the homologous to macaque IT cortex) and plotting the slope distribution as was done in monkey neurophysiology, they plotted the median slopes within LO searchlight clusters and rank-ordered them according to the classification accuracy of the clusters. From this result, it appeared that the average slope across all voxels was substantially lower than 0.5 and lower than what was obtained from monkey neurophysiology. This suggests that averaging may not occur in individual fMRI voxels, or at the very least, it does not provide definitive evidence showing the presence of averaging across individual fMRI voxels in LO. Thus, the simple correspondence of averaging at the unit level and at the population level has not been established in fMRI. We have previously reported averaging in LO in fMRI response patterns^7^. The first goal of the present study is to reexamine this data and test if averaging is indeed present in individual fMRI voxels. If it does, we will further test if voxels showing better averaging in response amplitude may exhibit better response pattern averaging at the population level.

Convolutional neural networks (CNNs) are currently considered as one of the best models of primate vision, achieving human-level performance in object recognition tasks and showing correspondences in object representation with the primate ventral visual system^12–14^. Meanwhile, there still exist large discrepancies in visual representation and performance between the CNNs and the primate brain^15^, with CNNs only able to account for about 60% of the representational variance seen in primate high-level vision^14,16–19^. While CNNs are fully image computable and accessible, they are also “blackboxes”—extremely complex models with millions or even hundreds of millions of free parameters whose general operating principles at the algorithmic level^20^ remain poorly understood^21^.

Because CNNs are trained with natural images containing a single target object appearing in a natural scene, it is unclear that objects are represented as distinctive units of visual processing and that an averaging relationship for representing multiple objects would automatically emerge. Moreover, neural averaging is similar to divisive normalization previously proposed to explain attentional effects in early visual areas^22–24^. Such a normalization process involves dividing the response of a neuron by a factor that includes a weighted sum of the activity of a pool of neurons through feedforward, lateral or feedback connections. Given that some of the well-known CNNs have no lateral or feedback connections, such as Alexnet^25^, VGG-16^26^, Googlenet^27^ and Resnet-50^28^, these CNNs may not show response averaging for representing multiple objects. Nonetheless, by assessing the averaged slope across CNN unit responses, Jacob et al.^29^ reported that higher layers of VGG16 exhibited averaging similar to that of macaque IT neurons. Because the slopes across all CNN units were averaged, this analysis does not tell us what the slope distribution across units is like and whether individual units indeed exhibit responses similar to those found in the primate brain. Meanwhile, averaging at the response pattern level has never been examined in CNNs. At the pattern level, if a large number of units fail to show averaging, even when the averaged slope from all the units is still pretty close to an averaging response, the pattern would deviate significantly from averaging. Thus, pattern analysis provides a more sensitive way of measuring averaging than the single unit slope analysis. Understanding the relationship between single and paired objects in CNNs is critical if we want to better understand the nature of visual object representations in CNNs and whether CNNs represent visual objects similarly as the primate brain. To do so, here we examined single unit and population responses from the higher layers of four CNNs pretrained for object categorization with varying architecture and depth. We additionally examined the higher layers of a CNN with recurrent processing to test whether averaging at both the single unit and population level may emerge when feedback connections are present in a network. As with the fMRI data, we also examined the relationship between unit and population responses by testing whether units showing better averaging in response amplitude would exhibit better pattern averaging at the population level.

## Results

**Figure 1:**
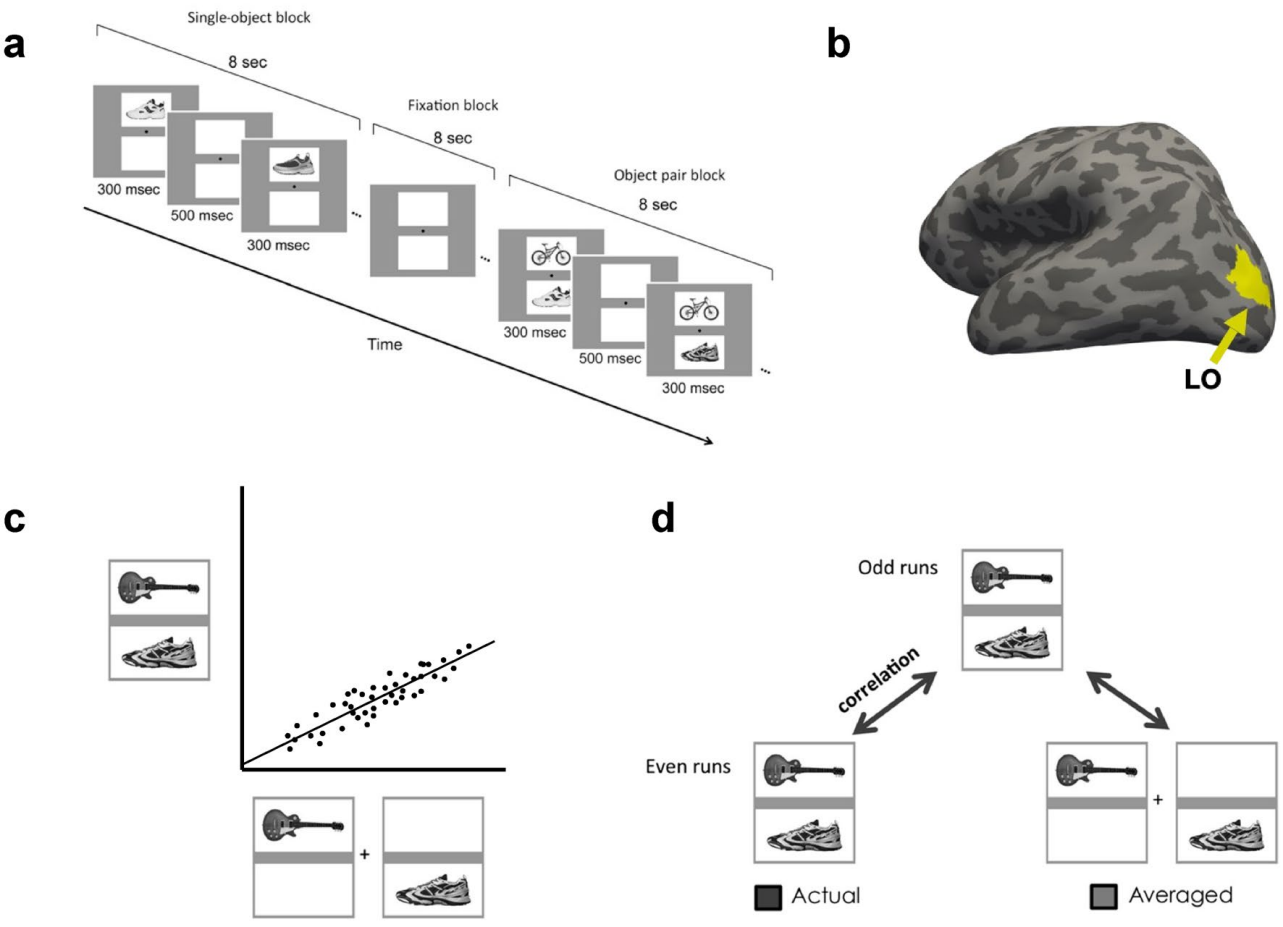
Experimental and analysis details. (**a**) Example trials of the main experiment. In single-object blocks, participants viewed a sequential presentation of single objects (different exemplars) from the same category at a fixed spatial location. In object pair blocks, they viewed a sequential presentation of object pairs from two different categories. The location of the objects from each of the two categories was fixed within a block. The task was to detect a 1-back repetition of the same object. In object pair blocks, the repetition could occur in either object category. The repetition occurred twice in each block. (**b**) Inflated brain surface from a representative participant showing LO. (**c**) A schematic illustration of the unit response analysis. We extracted, across all the included fMRI voxels/CNN units, the slope of the linear regression between an fMRI voxel/CNN unit’s response to a pair of objects and its summed response to the corresponding single objects shown in isolation. (**d**) A schematic illustration of the population response analysis. For LO, an object pair was correlated with itself across the odd and even halves of the runs (actual) and with the average of its constituent objects shown in isolation across the odd and even halves of the runs (average). For CNNs, because there is no noise, the object pair was simply correlated with the average of its constituent objects in isolation.

In this study, we analyzed a previous fMRI data set^7^ where participants viewed blocks of images showing either single object or object pairs selected from four different object categories (bicycle, couch, guitar, or shoe) and performed a 1-back repetition detection task (Fig. 1a). We selected the 75 most reliable voxels from LO in our analysis to equate the number of voxels from each participant and to increase power^30^. However, all results remained virtually identical when we included all voxels from LO. This indicates the stability and robustness of the results which did not depend on including the most reliable voxels.

We also analyzed the higher layers from five CNNs pre-trained using ImageNet images^31^ to perform object categorization. The CNNs examined included both shallower networks, Alexnet^25^ and VGG-19^26^, and deeper networks, Googlenet^27^ and Resnet-50^28^. We also included a recurrent network, Cornet-S, that has been shown to capture the recurrent processing in macaque IT cortex with a shallower structure and argued to be one of the current best models of the primate ventral visual system^18,32^. We further analyzed three versions of Resnet-50 trained on stylized versions of ImageNet images^33^ to examine how reducing texture bias and increasing object shape processing in a CNN would impact its representation of multiple objects. For all CNNs, we created a comparable set of activation patterns as the brain data. In the fMRI experiment, objects were presented within a white rectangle on a gray background. It is possible that this gray background could affect object averaging as CNNs wouldn’t “filter out” the gray background as human participants would. We thus examined CNN responses to objects on both the gray and white backgrounds.

### Evaluating the unit response to single and paired objects

In this analysis, we extracted, across all the included fMRI voxels/CNN units, the slope of the linear regression between an fMRI voxel/CNN unit’s response to a pair of objects and its summed response to the corresponding single objects shown in isolation. We averaged the slopes across all the human participants. The average slope should be 0.5 if the single fMRI voxel/CNN unit response to an object pair can be predicted by the average response of the corresponding objects shown alone. In a previous single-cell analysis of monkey IT, a slope of about 0.55 was reported^1^. We thus compared our slope results to both 0.5 and 0.55 as baselines. Following Jacob et al.^29^, we only selected CNN units that showed non-zero responses to objects at both locations.

#### Human LO

**Figure 2:**
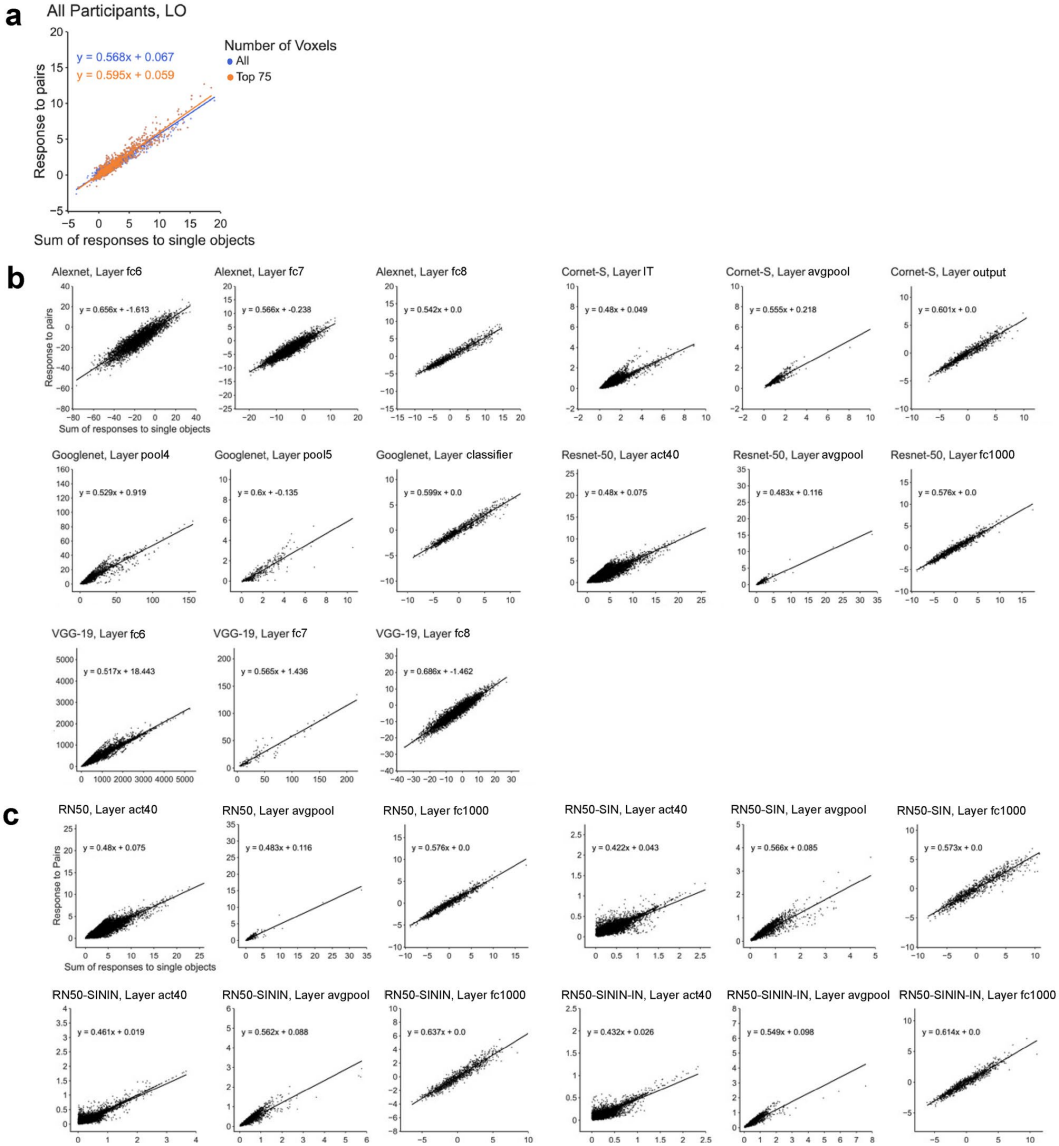
Single unit response amplitude distribution results. (**a**) Single voxel response to object pairs plotted against the sum of the individual object responses in LO for all of the participants for both the top 75 most reliable voxels and all the voxels. Units are in beta weights. (**b**) Responses of the five CNNs (pretrained on the original ImageNet images) to paired and single objects on gray background. (**c**) Responses of Resnet-50 to paired and single objects on gray background. Resnet-50 was pretrained either with the original ImageNet images (RN50-IN), the stylized ImageNet Images (RN50-SIN), both the original and the stylized ImageNet Images (RN50-SININ), or both sets of images, and then fine-tuned with the stylized ImageNet images (RN50-SININ-IN).

**Figure 3:**
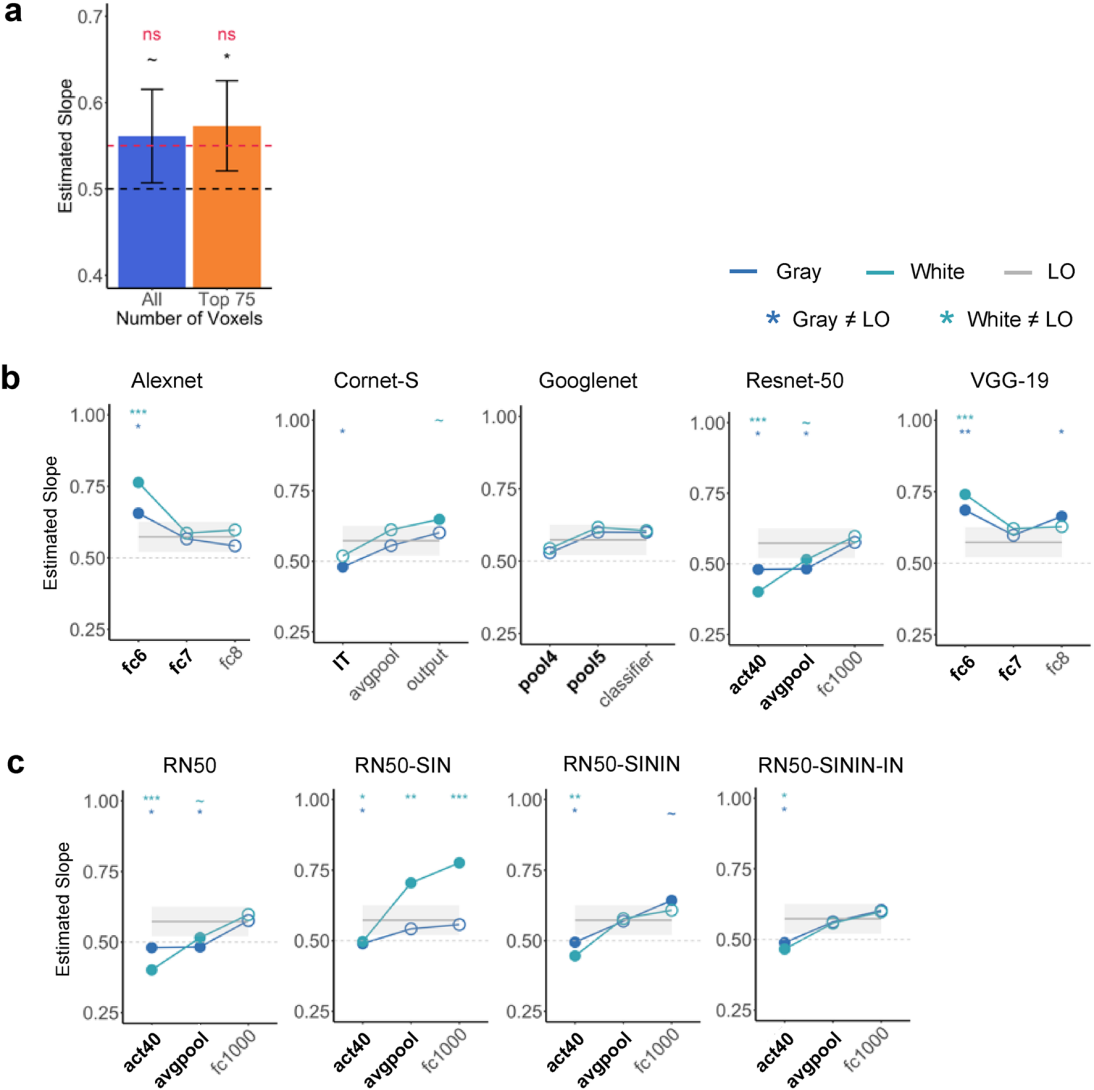
Single unit response summary results. (**a**) The averaged slope across all participants for both the top 75 most reliable LO voxels and for all the LO voxels. The average slope is compared to the slope of a perfect averaging (0.5) as well as the slope reported in single cells in macaque IT (0.55). (**b**) Comparing LO slope with those of 5 CNNs with the objects appearing on both the gray and white backgrounds. Bold layers were those that showed best correspondence with LO as shown by Xu and Vaziri-Pashkam^14^. For each layer, significant values for pairwise t tests against the slope of LO are marked with asterisks at the top of the plot. (**c**) Comparing LO slope with those of Resnet-50 under different training regimes. Resnet-50 was pretrained either with the original ImageNet images (RN50-IN), the stylized ImageNet Images (RN50-SIN), both the original and the stylized ImageNet Images (RN50-SININ), or both sets of images, and then fine-tuned with the stylized ImageNet images (RN50-SININ-IN). All t tests were corrected for multiple comparisons using the Benjamini–Hochberg method for 2 comparisons in LO, and for 6 comparisons (3 layers × 2 background colors) for each CNN. Error bars and ribbons represent the between-subjects 95% confidence interval of the mean. ~ 0.05 < p < 0.10, *p < 0.05, **p < 0.01, ***p < 0.001.

The average slope for all participants when including the 75 most reliable voxels and all the voxels were 0.573 and 0.561, respectively (Fig. 3a). These average slopes did not differ from the reported IT neuron slope of 0.55 (*t*(9) = 0.866, *p* = 0.694, *d* = 0.273, for the 75 most reliable voxels; and *t*(9) = 0.406, *p* = 0.694, *d* = 0.128, for all the voxels; corrected for multiple comparisons using the Benjamini–Hochberg method for two comparisons, see^34^). These slopes, however, did deviate from the perfect averaging slope of 0.5 (*t*(9) = 2.741, *p* = 0.046, *d* = 0.867, for the 75 most reliable voxels; and *t*(9) = 2.22,* p* = 0.054, *d* = 0.701 for all the voxels; corrected). Inspection of the distribution of the voxel response aggregated across all participants revealed a distribution around the line of best fit, similar to a normal distribution and the distribution seen in the IT neuron responses (Fig. 2a). Overall, we observed averaging in single fMRI voxels in human LO comparable to that of single neurons in macaque IT.

#### CNNs

When the slopes from the sampled CNN layers were directly tested against the slope obtained from the human LO for the 75 most reliable voxels, 8 out of the 15 (53%) examined layers did not differ from LO for either the white or gray background images (*ts* < 2.05, *ps* > 0.11; all others, *ts* > 2.82, *ps* < 0.06; see the asterisks marking the significance levels on Fig. 3b; all pairwise comparisons reported here and below were corrected for multiple comparisons using the Benjamini–Hochberg method). When we further examined the layers that showed the greatest correspondence to LO as reported in a previous study^14^, 4 out of the 9 (44%) layers were not significantly different from LO (see the layers marked with bold font in Fig. 3b). There was little effect of image background (i.e., whether the images appeared on the white or gray backgrounds), with only 3 out the 15 (20%) layers showing a discrepancy when comparing with LO (i.e., with performance on one background being similar and performance on the other background being different from that of LO). For the other 12 layers, performance (as compared to LO) did not differ across the two backgrounds. Overall, among the CNNs tested, Googlenet best resembled the human LO, with all of its sampled layers showing no significant difference from LO. Interestingly, the recurrent network examined, Cornet-S, did not seem to behave differently from the other networks.

In Jacob et al.^29^, there was a noticeable improvement in unit averaging from lower to higher layers. Here we found that, except for Googlenet, the other CNNs all showed an improvement between the first two examined layers. Resnet-50 additionally showed an improvement between the last two examined higher layers.

Close inspection revealed that the distribution of the CNN unit responses to object pair and single objects varied greatly across the examined CNN layers, with some showing a distribution resembling that of a normal distribution and those of LO voxels and IT neurons, but others deviating greatly from such a distribution even when the average slope was close to 0.5 or 0.55 (Fig. 2b). For example, although layer fc1000 in Resnet-50 and layer pool4 in Googlenet had slopes close to 0.5 and 0.55 (0.576 and 0.529, respectively), Resnet-50 had an approximately normal distribution while Googlenet does not. Additionally, although layer fc1000 in Resnet-50 and layer fc6 in Alexnet had approximately normal distributions, their slopes were very different from each other (0.576 and 0.656, respectively). This suggested that the representation of multiple objects in some higher CNN layers was different from that of the human LO and macaque IT.

### Evaluating the pattern response to single and paired objects

In this analysis, we wanted to replicate the existence of averaging in fMRI response patterns as reported in Jeong and Xu^7^ such that response patterns for an object pair may be predicted by the average pattern of the constituent objects shown in isolation. We also tested whether such averaging existed in CNN unit response patterns. To analyze the fMRI pattern responses, as in Jeong and Xu^7^, we conducted a split-half analysis to account for measurement noise (as correlation could never reach 1 even for the same condition across different halves of the data due to the presence of measurement noise in fMRI data). Specifically, we divided the runs into even and odd halves and averaged the response patterns for each condition within each half of the runs. We then correlated the response patterns of the same object pairs across even and odd halves (actual pair correlation) and correlated the response patterns of an object pair from one half and the average response pattern of the constituent single objects in the other half (average pair correlation). Finally, using the actual pair correlation as a baseline, we derived a noise-normalized correlation by dividing the average pair correlation by the actual pair correlation. If averaging existed in response patterns, then the response pattern of an object pair would be as similar to itself in the other half of the runs as it would be to the averaged response patterns of the single objects in the other half of the runs. We would then have a noise-normalized correlation no different from 1. For the CNNs, because there was no noise, we simply correlated the response pattern of a pair of objects with the average response pattern of the constituent single objects. We then compared this correlation to that of LO. We additionally assessed whether a weighted average of single objects would improve pattern averaging. We did so by systematically varying the contribution of the two single objects’ response patterns from 0 to 100%, with 10% increments in between.

#### Human LO

**Figure 4:**
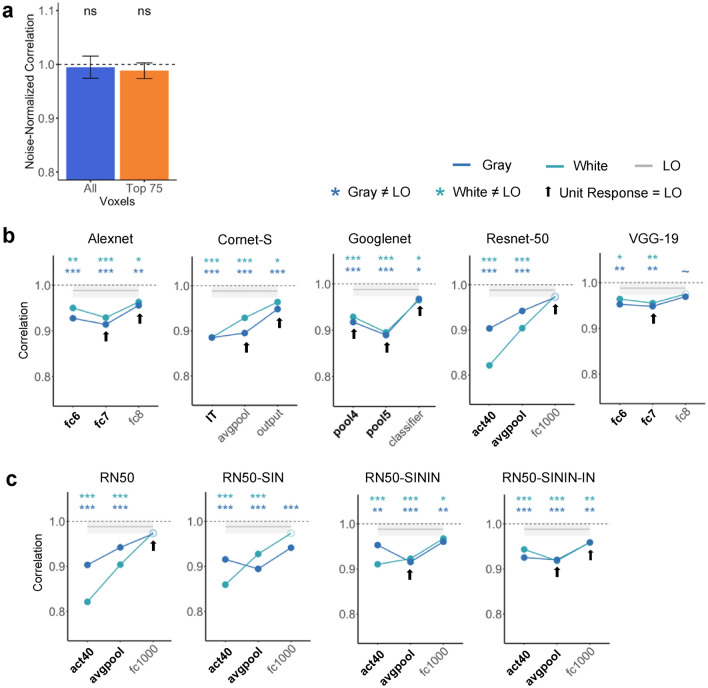
Pattern response results. (**a**) Noise-normalized correlation between the pattern response to an object pair and the average pattern response to the individual objects in LO for the top 75 most reliable voxels and all the voxels. A one-sided t-test was conducted comparing the correlations to 1. (**b**) Comparing pattern averaging in LO with those of five CNNs with the objects appearing on both the gray and white backgrounds. Bold layers were those that showed best correspondence with LO as shown by Xu and Vaziri-Pashkam^14^. For each layer, significant values for pairwise t tests against LO are marked with asterisks at the top of the plot. Layers showing comparable averaging in unit response amplitude to that of LO are marked with an arrow. (**c**) Comparing pattern averaging in LO with that of Resnet-50 under different training regimes. Resnet-50 was pretrained either with the original ImageNet images (RN50-IN), the stylized ImageNet Images (RN50-SIN), both the original and the stylized ImageNet Images (RN50-SININ), or both sets of images, and then fine-tuned with the stylized ImageNet images (RN50-SININ-IN). All t tests were corrected for multiple comparisons using the Benjamini–Hochberg method for 2 comparisons in LO, and for 6 comparisons (3 layers × 2 background colors) for each CNN. Error bars and ribbons represent the between-subjects 95% confidence interval of the mean. ~ 0.05 < p < 0.10, *p < 0.05, **p < 0.01, ***p < 0.001.

**Figure 5:**
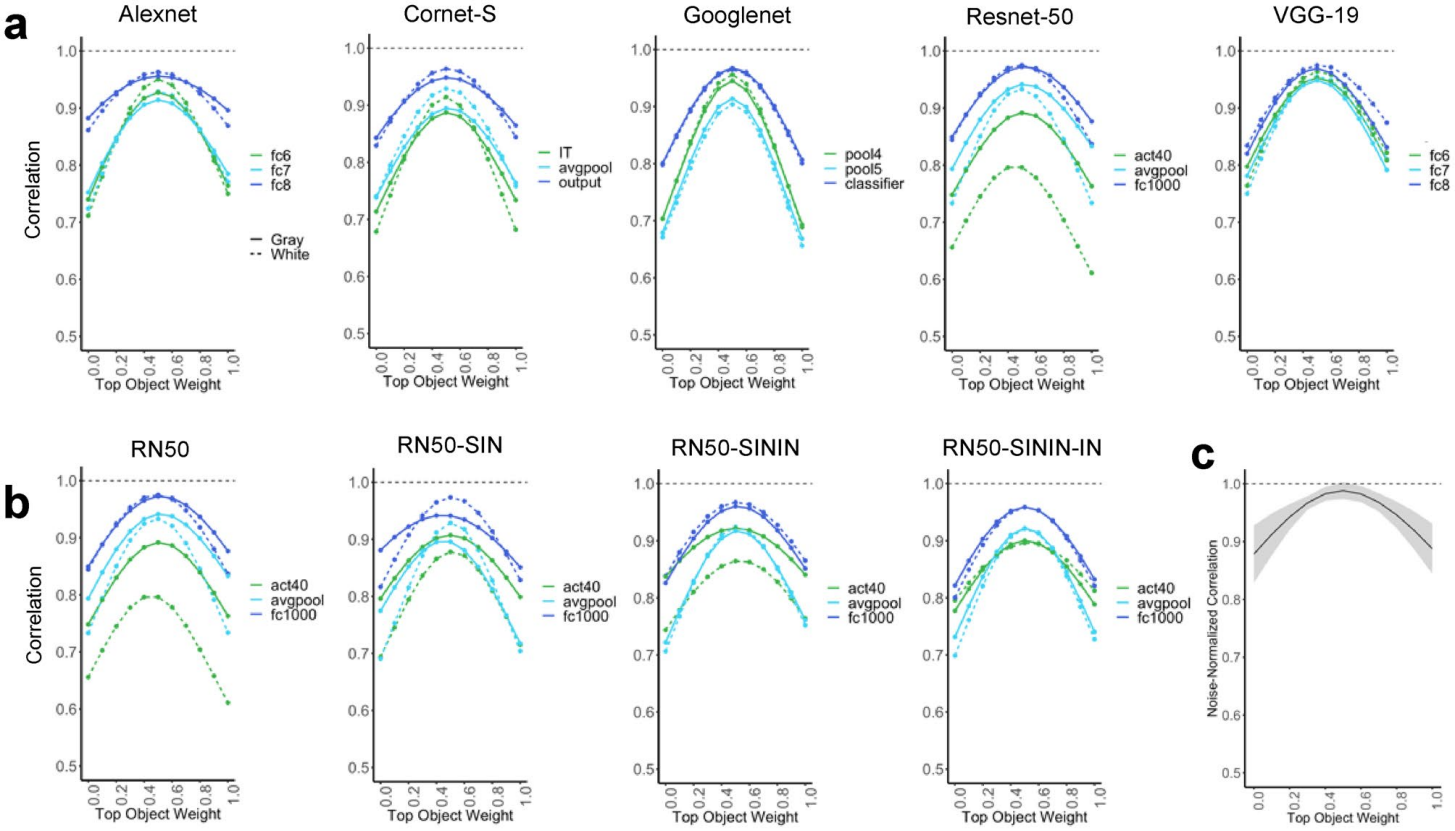
Pattern averaging results with a weighted average of the two single objects, where the weight of each object varies between 0 and 1 with increments of 0.1. (**a**) Weighted pattern averaging for the five CNNs trained on the original ImageNet images. (**b**) Weighted pattern averaging for Resnet-50. Resnet-50 was pretrained either with the original ImageNet images (RN50-IN), the stylized ImageNet Images (RN50-SIN), both the original and the stylized ImageNet Images (RN50-SININ), or both sets of images, and then fine-tuned with the stylized ImageNet images (RN50-SININ-IN). (**c**) Weighted pattern averaging for LO including all the voxels.

In LO, the noise-normalized correlation did not differ from 1 for neither the Top 75 voxels (*t*(9) = 1.588, *p* = 0.146, *d* = 0.502; one-tailed as only comparison in one direction was meaningful here) nor all voxels (*t*(9) = 0.498, *p* = 0.315, *d* = 0.157; one-tailed) (corrected for two comparisons) (Fig. 4a). Additional analyses showed that weighing the two constituent objects equally yielded the best averaging prediction (Fig. 5c). These results replicated those of Jeong and Xu^7^ and showed the existence of averaging in fMRI response patterns in human LO.

#### CNNs

Overall, all sampled CNN layers showed a correlation in pattern response greater than 0.8. However, with the exception of the last sampled layer in Resnet-50, for all others, the correlation was significantly less than the noise-normalized correlation of the top 75 voxels in LO regardless of the background color (see the asterisks marking the significance levels in Fig. 4b). This suggests that pattern averaging of single object responses could not fully predict that of the paired objects in most of the sampled CNN layers, different from the results obtained in human LO (Fig. 4b). This was true even for layers showing strong averaging in single unit response amplitudes as shown in Fig. 3b and marked on Fig. 4b, indicating a discrepancy between unit and pattern responses in these CNN layers. This was also true for Cornet-S, the recurrent network that included feedback connections among the units to better model the primate visual system. Of all the CNN layers sampled, only the final layer of Resnet-50 exhibited pattern correlation and single unit responses matching those of LO (see Figs. 3b, 4b). That being said, both Cornet-S and Resnet-50 showed an improvement in averaging between the first two sampled layers, and all CNNs showed an improvement between the last two sampled layers, although such improvement was still not sufficient to reach the performance of LO. Additional analyses showed that, similar to LO, an equally weighted averaging of both constituent objects yielded the best pattern prediction (Fig. 5a).

### Evaluating the relationship between unit response and pattern response

In this analysis, we wanted to gain a better understanding of the relationship between unit and population responses and why there might be a discrepancy between unit and pattern responses in CNNs. We evaluated whether single units showing better averaging in response amplitude also show better averaging in response pattern. We divided LO voxels/CNN units into two groups by whether the slope in response amplitude averaging for the single voxel/CNN unit was near or far from 0.5, with the *near* condition defined as having a slope between 0.45 and 0.55 and the *far* condition defined as having a slope less than 0.45 or greater than 0.55. We then assessed averaging in response patterns in the near and far groups of voxels and units separately. We also noted the percentage of voxels/CNN units in the two groups.

#### Human LO

**Figure 6:**
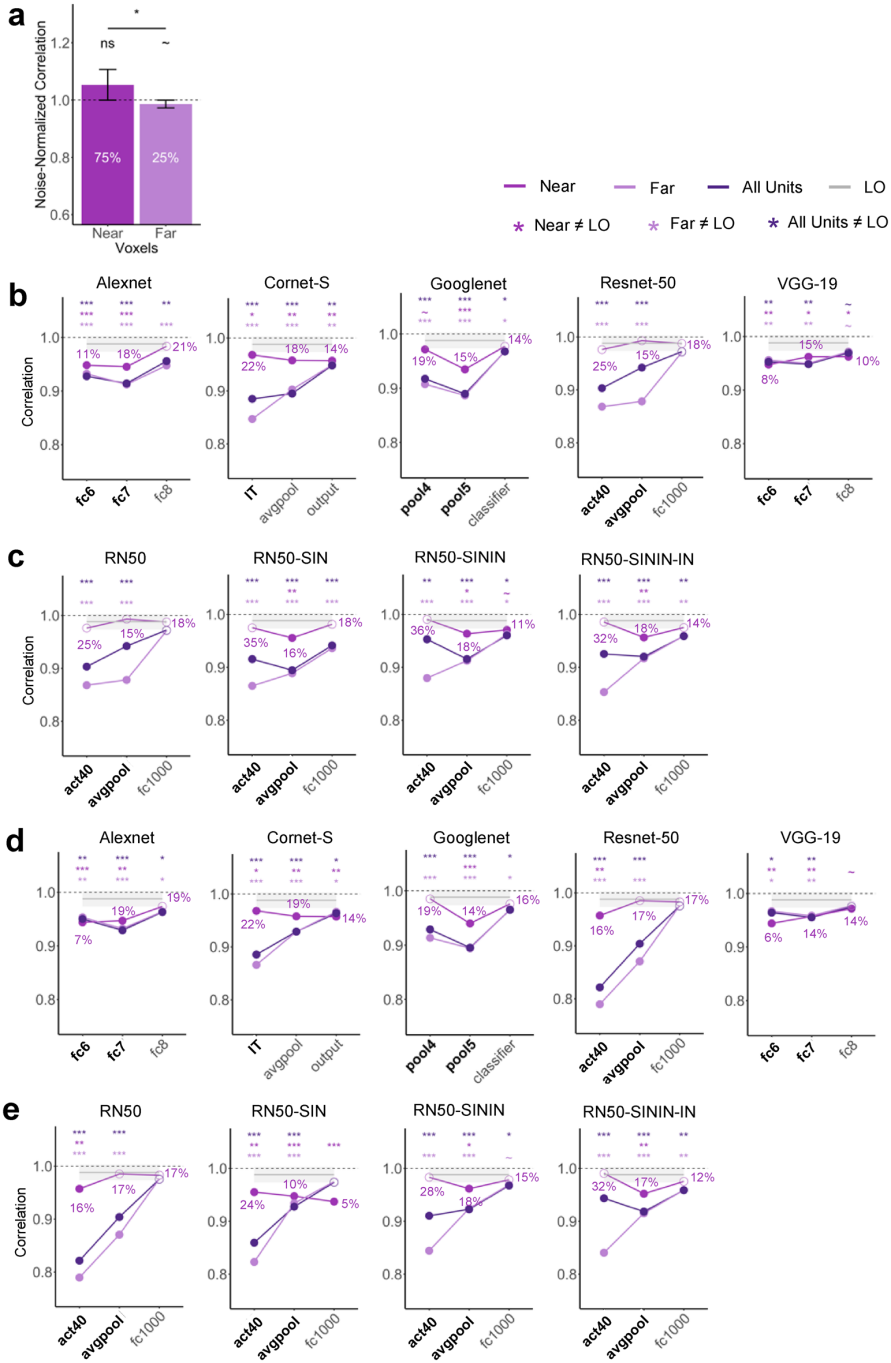
Relationship between unit and pattern responses. (**a**) Noise-normalized correlation between the pattern response to the pair and the average pattern response to the individual objects in LO for voxels that have slope near 0.5 (0.45 < slope < 0.55) and for voxels that have slope far from 0.5 (slope < 0.45 or slope > 0.55). The percentage of voxels considered to be near or far from slope 0.5 are marked. A one-sided t-test was conducted comparing the correlations to 1. (**b**) CNN pattern averaging results for images with gray background, separately for CNN units with slope near 0.5, units with slope far from 0.5, and all units, all compared with pattern averaging from LO. The percentages of units near slope 0.5 are marked. Bold layers were those that showed best correspondence with LO as shown by Xu and Vaziri-Pashkam^14^. For each layer, significant values for pairwise t tests against the slope of LO are marked with asterisks at the top of the plot. (**c**) For Resnet-50 under different training regimes, CNN pattern averaging results for images with gray background, separately for CNN units with slope near 0.5, units with slope far from 0.5, and all units, all compared with pattern averaging from LO. Resnet-50 was pretrained either with the original ImageNet images (RN50-IN), the stylized ImageNet Images (RN50-SIN), both the original and the stylized ImageNet Images (RN50-SININ), or both sets of images, and then fine-tuned with the stylized ImageNet images (RN50-SININ-IN). (**d**) CNN pattern averaging results for images with white background, separately for the three groups of units as in (**b**). (**e**) Resnet-50 pattern averaging results for images with white background, separately for the three groups of units as in c. All t tests were corrected for multiple comparisons using the Benjamini–Hochberg method for 2 comparisons in LO, and for 9 comparisons (3 layers × 3 groups of units) in each CNN. Error bars and ribbons represent the between-subjects 95% confidence interval of the mean. ~ 0.05 < p < 0.10, *p < 0.05, **p < 0.01, ***p < 0.001.

We included all LO voxels in this analysis. Overall, approximately 75% of the total voxels across all of the participants had a slope near 0.5 (Fig. 6a). Such a distribution is consistent with the voxel distribution seen in Fig. 6a. While there was no difference between the noise-normalized pattern correlation and 1 for the near voxels (*t*(9) = 1.942, *p* = 0.958, *d* = 0.614; corrected), the difference was marginally significant for the far voxels (*t*(9) = 2.029, *p* = 0.073, *d* = 0.642; corrected), with a significant difference between the near and far voxels (*t*(9) = 2.52, *p* = 0.033, *d* = 1.06). In human LO, voxels with a better averaging response in response amplitude thus also showed better averaging in response patterns.

#### CNNs

In CNNs, depending on the layer and network, only 5–35% of the total units had a slope near 0.5 (Fig. 6b–e). This distribution was very different from that of LO where 75% of the voxels had a slope near 0.5. This confirmed the observation made earlier when we inspected the distribution of the unit responses (see Fig. 2a,b) and once again showed that extracting the slope across all the units could significantly obscure differences in the overall unit response distributions. For the CNN units that were near 0.5, we did see improvement in pattern averaging, with 10/14 (71.4%) of the layers that did not previously show strong pattern averaging now showing increased averaging regardless of image background (see the asterisks marking the significance levels in Fig. 6b,d). However, only 3/14 (21.4%) of these layers now showed averaging no different from LO. Thus, better averaging in unit response amplitudes improved but rarely led to full pattern averaging similar to that found in the human LO.

### Evaluating the effect of shape training on unit and pattern normalization

Because CNNs trained on ImageNet images are biased toward texture rather than shape processing^33^, we evaluated whether CNNs biased towards shape processing may exhibit more LO like averaging in both response amplitude and response patterns. We tested 3 versions of Resnet-50 trained with stylized ImageNet images that emphasizes shape processing: Resnet-50 trained on Stylized ImageNet alone (RN50-SIN), Resnet-50 trained on both Original and Stylized ImageNet, and Resnet-50 trained on both Original and Stylized ImageNet (RN50-SININ), with fine-tuning on ImageNet (RN50-SININ-IN)^33^.

At the single unit level, when we compared the slopes of Resnet-50 trained on just the Original ImageNet images with those that were trained on Stylized ImageNet images, we saw the biggest improvement occurred in RN50-SININ-IN (Fig. 3c). The original Resnet-50 was only comparable to LO in the final layer, but RN50-SININ-IN was comparable in the last two layers. Meanwhile, at the population level, such shape-training did not improve averaging in response patterns (Fig. 4c). Thus, training Resnet-50 to emphasize shape rather than texture processing showed some improvement at the single unit level, but not at the population level.

## Discussion

Previous monkey neurophysiology, human fMRI and CNN studies have reported that neural/CNN responses to a pair of unrelated objects can be well predicted by the average responses to each constituent object shown in isolation, allowing primate vision to recover the identity of an object regardless of whether or not other objects are encoded concurrently. Such an averaging relationship has been found at the single unit level in the slope of response amplitudes of macaque IT neurons and CNN units to paired and single objects^1,29,35^ and at the population level in response patterns of fMRI voxels in the human ventral object processing region^3–7^. Although it is assumed that averaging at the single unit and population levels must reflect the same underlying process, the only human fMRI study that has examined this appeared to indicate the absence of such averaging across all the LO voxels^7^. By evaluating the slope of single unit responses, object-categorization-trained CNN units are also shown to exhibit the same averaging responses as the macaque IT neurons^29^. However, averaging the slope across units ignores the overall response distribution, potentially obscuring important differences in how CNNs and the primate brain represent multiple objects. To address these questions, here we first tested whether averaging at the single unit level similar to that found in IT neurons may exist in single fMRI voxels. Conversely, we tested whether averaging in response patterns as observed in human fMRI studies may be present in CNN unit response patterns. We then examined whether voxels/units showing better averaging in response amplitude exhibit better response pattern averaging at the population level. To accomplish these goals, we analyzed fMRI responses from the human LO from an existing data set^7^. We also examined responses from five CNNs pretrained for object categorization, including Alexnet, Cornet-S, Googlenet, Resnet-50, and VGG-19.

In human LO, replicating Jeong and Xu^7^, we observed averaging in fMRI response pattern between paired and single objects. Unlike MacEvoy and Epstein^3^, we also observed averaging in the response amplitude of single fMRI voxels across LO similar to that reported in single neuron recordings in macaque IT^1^. Specifically, across all the included fMRI voxels, the slope of the linear regression between an fMRI voxel’s response to a pair of objects and its summed response to the single objects was indistinguishable from that of the IT neurons. The same results were obtained whether the top 75 most reliable voxels or all the voxels were included, demonstrating the robustness of the effect. Thus, by directly applying the method used in neurophysiology, we show that the same result reported in monkey neurophysiology studies is present in fMRI voxels even though fMRI is an indirect measure of neural activity with each voxel containing millions of neurons. Further analyses revealed that about 75% of LO voxels showed a slope close to 0.5 when the slope was measured within each voxel. As expected, voxels showing better averaging in response amplitude (i.e., with a slope close to 0.5) also exhibited better averaging in response patterns.

CNNs have been considered by some as the current best models of the primate ventral visual system^32,36^. Meanwhile, large discrepancies in object representation also exist between the primate brain and CNNs^14,15,19,33,37^. In a previous study, Jacob et al.^29^ observed averaging in unit responses in the higher layers of VGG-16 pretrained for object categorization. Here in the higher layers of five CNNs with varying architecture, processing depth, and the presence/absence of recurrent processing, we observed in about half of the sample layers response amplitude averaging in the CNN units comparable to those found in the human LO (i.e., the slope across all the included units did not differ from that of LO). Except for Googlenet, which showed equally good averaging as the human LO in all the sampled layers, for the other four CNNs, there was a general increase in averaging from the lower to the higher sampled layers. Overall, these results replicated and extended the results of Jacob et al.^29^ and showed that averaging, as assessed by the response amplitude slope across all the units, exists in CNN unit responses for representing a pair of objects at the higher levels of CNN visual processing.

Despite the prevalence of averaging in CNN single unit responses, except for the final layers of Resnet-50, no CNN higher layers showed averaging in response patterns comparable to that of the human LO regardless of differences in the exact architecture, depth, and presence/absence of recurrent processing. For example, the recurrent CNN we tested, Cornet-S, which was designed to closely model ventral visual processing, did not outperform the other CNNs. This was also true for layers showing good correspondence with LO in object representational structure as reported in a separate study (Ref.^14^; note that the final sampled layer of Resnet-50, which showed the best pattern averaging as LO, did not show the best correspondence with LO in representational structure). There is thus a discrepancy between unit and pattern responses in CNNs. Further investigation revealed that, in many of the higher CNN layers sampled, the distribution of unit responses to paired and single objects did not resemble that of the human LO or macaque IT. In fact, when we divided up the units into two groups based on whether the response amplitude of a unit showed good or bad averaging (i.e., whether the slope was near or far from 0.5), we found that only a minority of the CNN units showed good averaging (5–35% depending on the layer) compared to the majority of the LO voxels that showed good averaging (75%). Thus, extracting the slope across all the units and comparing that with the brain obscured significant distributional differences between the brain and CNNs, leading to the impression that CNNs represent paired objects in a similar way as the primate brain, but in fact the majority do not. At the pattern level, when a large number of units failed to show averaging, even though the averaged slope from all the units would not be far from 0.5, the pattern would deviate significantly from averaging. In this regard, pattern analysis is a more sensitive way of measuring averaging than the single unit slope analysis. Like the LO voxels, CNN units with good averaging also tended to show better averaging in response patterns, but this rarely led to full pattern averaging similar to that found in the human LO.

In the present study, we also considered three factors that may impact the results. In the fMRI experiment, objects were presented within a white rectangle on a gray background. It is possible that this gray background could affect object averaging as CNNs wouldn’t “filter out” the gray background as human participants would. We thus examined CNN responses to objects on both the gray and white backgrounds, but found very little effect of background on CNN unit response amplitude or response pattern. In the main analyses, we only considered a simple average in which the response of each object contributed equally to the prediction of the response of the paired objects. In additional analyses, we also weighed the two objects differently but found that an equal weighting best predicted the response of object pairs from single objects in both the human LO and higher CNN layers. Lastly, to account for the texture bias observed in CNNs, we examined responses from Resnet-50 trained with the stylized versions of ImageNet images that emphasized shape processing^33^. While we observed some improvement in averaging in single unit responses, we saw no improvement in pattern averaging. Such a CNN training regime thus does not appear to make the CNN representation of the paired objects to be more brain-like.

Overall, we found that averaging existed in both single fMRI voxels and voxel population responses in the human LO, with better averaging at the single voxels leading to better averaging in fMRI response patterns. Although CNNs exhibited averaging at the unit level when the slope of response amplitudes for single and pair objects were considered across all the units, detailed investigation revealed that unit response distribution in most cases did not resemble those of the human LO or macaque IT. Consequently, averaging at the population level for the CNN units did not match that of LO. The whole is thus not equal to the average of its parts in CNNs.

Both monkey neurophysiology and human fMRI studies have shown that simple averaging no longer holds when two objects interact with each other, such as being connected^37^ or forming familiar and meaningful pairs (e.g., a water jug and a cup^38^). It would be interesting to include object pairs during CNN training and test whether the inclusion of these object pairs would further increase their interaction in CNN representation. Meanwhile, given that the specific object pairs we used here were unlikely to have appeared together in the CNN training data, our observed interactions in their representations when they were shown together still indicates that object representations formed in the current CNNs are likely very different from those in the primate brain.

Instead of examining CNNs trained for object classification, one could test averaging in CNNs trained for scene classification, as the training images in this case naturally contain multiple objects embedded in a co-occurring background. Consequently, averaging for single and paired objects may be present in such networks. In a human fMRI study, MacEvoy and Epstein^4^ reported that while fMRI response patterns evoked by scenes in LO could be well predicted by the average patterns elicited by the signature objects in the scene, this was not the case in the parahippocampal place area, a brain region specialized for scene processing^39^. In other words, specialization in scene processing appears to result in an interaction, rather than the independent representations, of the objects in a scene. This predicts that scene-trained CNNs would not exhibit averaging for single and paired objects. However, we recently reported that object- and scene-trained CNNs represent the identities and the configuration of the objects in a scene in a similar way^40^, suggesting that scene-trained CNNs are not necessarily more sensitive to object configurations than object-trained CNNs. That said, it could still be useful for future studies to directly compare averaging in scene-trained CNNs and in object-trained CNNs.

It may be argued, however, that the pattern averaging results from CNNs still resemble those of the brain, even when there were significant differences. Given that the exact same visual features were present in an object pair and in single objects, a high correlation in CNN representations between the actual pair and the averaged pair was not surprising. However, while in the human brain, the paired object representation was equivalent to the average representation of the single objects shown alone, this was not the case in CNNs. Here, we are not interested in a “glass half empty vs half full” debate, but rather what the significant difference seen in CNNs would imply. As noted before, one of the hallmarks of primate high-level vision is its ability to extract object identity features among changes in non-identity features and form transformation-tolerant object representations^9–11^. This allows us to rapidly recognize an object under different viewing conditions. One aspect of this is clutter tolerance, the ability to form the same object representation regardless of whether it is shown alone or with another object^41^. Our results showed that in CNNs, object representations interacted with each other when objects were shown together and differed significantly from when objects were shown alone. In other words, CNNs distorted an object’s representation when it was shown with another object. Such a distortion would make object representations formed in CNNs context dependent and could well underlie the two major drawbacks current CNNs face: the requirement of large training data and the inability to generalize to new objects not included in training^15^. Our study thus identifies a potential limitation in the computational algorithms employed by current CNNs and suggests that CNNs do not form the same kind of transformation-tolerant visual object representations as the human brain. This echoes the finding from another recent study of ours which shows that, unlike human vision, CNNs do not maintain the object representational structure at higher levels of visual processing when objects undergo transformations involving changes such as position or size^42^.

In the primate brain, neural averaging has been linked to a normalization process that involves dividing the response of a neuron by a factor that includes a weighted sum of the activity of a pool of neurons through feedforward, lateral or feedback connections^22–24^. Future CNN architecture development may explicitly impose such a circuit property at higher levels of visual processing. It may also be possible to impose brain-like object averaging in CNN unit responses within the current architecture during training. Such manipulations may not only make CNNs exhibit brain-like averaging in object representations but may also help them overcome some of their current limitations, making CNNs both better models for the primate brain and better models for object recognition.

## Materials and methods

In this study we reanalyzed data from an existing fMRI data set^7^ where participants viewed both object pairs and their constituent single objects in a 1-back repetition detection task. We extracted fMRI responses from lateral occipital cortex (LO), a higher ventral visual object processing region homologous to the macaque IT. We also extracted CNN unit responses to the same images from five CNNs pre-trained on object recognition using ImageNet images^30^. We examined both shallower networks, including Alexnet^25^ and VGG-19^26^, and deeper networks, including Googlenet^27^ and Resnet-50^28^. We also included a recurrent network, Cornet-S, that has been shown to capture the recurrent processing in macaque IT cortex with a shallower structure, and is argued to be one of the current best models of the primate ventral visual system^18,32^. As an additional test, because CNNs trained on ImageNet images are biased toward texture rather than shape processing, we evaluated Resnet-50 trained with stylized ImageNet images that emphasized shape processing^33^. We examined single unit responses in human LO fMRI voxels and in CNN higher layer units. We also examined population responses in human LO fMRI voxel response patterns and in CNN unit response patterns. We then compared single unit and population responses both within human LO and CNNs and between the two systems.

The details of the fMRI experiment has been reported in a previous publication^6^. They are summarized here for the readers’ convenience.

### Participants

Ten paid participants (eight women) took part in the study with informed consent. They all had normal or corrected-to-normal visual acuity, were right-handed, and between 18 and 35 years old (M = 29.33 years, SD = 3.08 years). One additional participant was tested but excluded from data analysis due to excessive head motion (> 3 mm). The study was approved by the institutional review board of Harvard University. All experiments were performed in accordance with relevant guidelines and regulations.

### Experimental design and procedures

#### Main experiment

Participants viewed blocks of images in the main experiment (Fig. 1). Each single-object block contained a sequential presentation of 10 images from the same object category either all above or all below the central fixation. Each object pair block contained a sequential presentation of two streams of 10 exemplars from two different categories with one always above and one always below the central fixation. Participants performed a 1-back repetition detection task and pressed a response key whenever they detected an immediate repetition of the exemplar. A repetition occurred twice in each block. In object pair blocks, the repetition occurred randomly in either the upper or lower location. The presentation order of the stimulus blocks and the presentation order of the exemplars within each block were randomly chosen. Four object categories (shoe, bike, guitar, and couch) with each containing 10 different exemplars were shown. All the exemplars from a given object category were shown in the same view and thus shared a similar outline. This allowed us to increase the difficulty of the 1-back task, thereby increasing participants’ attentional engagement on the task. This also resulted in objects from different categories to be more distinctive from each other. There were eight unique single-object blocks (4 object categories × 2 locations) and 12 unique object pair blocks (with all possible combinations of object categories and locations included).

Each exemplar image subtended approximately 5.5° × 2.8°. Two white square placeholders (7° × 4.7°), marking the two exemplar locations, were shown above and below the central fixation throughout a block of trials. The distance between the central fixation and the center of each placeholder was 3.2°. Each stimulus block lasted 8 s and contained 10 images, with each appearing 300 ms followed by a 500-ms blank interval. Fixation blocks, which lasted 8 s, were inserted at the beginning and end of the run and between each stimulus block. Each run contained 20 stimulus blocks, with each unique stimulus block appearing once, and 21 fixation blocks. Each participant was tested with 10 runs, each lasting 5 min 36 s. Participants’ eye movements during the main experiment were monitored with an EyeLink 1000 eye tracker to ensure proper central fixation.

#### LO localizer

Following Xu and Jeong^43^, to localize LO, participants viewed blocks of sequentially presented object and noise images (both subtended approximately 12° × 12°). Each object image contained four unique objects shown above, below, and to the left and right of the central fixation (the distance between the fixation and the center of each object was 4°). Gray-scaled photographs of everyday objects were used as the object stimuli. To prevent grouping between the objects, objects appeared on white placeholders (4.5° × 3.6°) that were visible throughout an object image block. Noise images were generated by phase-scrambling the object images used. Each block lasted 16 s and contained 20 images, with each image appearing for 500 ms followed by a 300-ms blank display. Participants were asked to detect the direction of a slight spatial jitter (either horizontal or vertical), which occurred randomly once in every 10 images. Eight object blocks and eight noise blocks were included in each run. Each participant was tested with two or three runs, each lasting 4 min 40 s.

### MRI methods

fMRI data were acquired from a Siemens (Erlangen, Germany) Tim Trio 3-T scanner at the Harvard Center for Brain Science (Cambridge, MA). Participants viewed images back-projected onto a screen at the rear of the scanner bore through an angled mirror mounted on the head coil. All experiments were controlled by an Apple MacBook Pro laptop running MATLAB (The MathWorks Natick, MA) with Psychtoolbox extensions^44^. For the anatomical images, high-resolution T1-weighted images were acquired (repetition time = 2200 ms, echo time = 1.54 ms, flip angle = 7°, 144 slices, matrix size = 256 × 256, and voxel size = 1 × 1 × 1 mm). Functional data in the main experiment and in the LO localizer were acquired using the same gradient-echo echo-planar T2*-weighted sequence (repetition time = 2000 ms, echo time = 30 ms, flip angle = 90°, 31 slices, matrix size = 72 × 72, voxel size = 3 × 3 × 3 mm, 168 volumes for the main experiment and 140 volumes for the LO localizer).

### Data analysis

#### fMRI data processing

fMRI data were analyzed using FreeSurfer (surfer.nmr.mgh.harvard.edu), FsFast^45^, and in-house MATLAB and Python codes. FMRI data preprocessing included 3D motion correction, slice timing correction and linear and quadratic trend removal. No smoothing was applied to the data. The first two volumes of all functional runs were also discarded. All the analysis for the main experiment was performed in the volume. The ROIs were selected on the surface and then projected back to the volume for further analysis.

LO was defined separately in each participant as a cluster of continuous voxels in the lateral occipital cortex showing higher activations to the object images than to the noise images (*p* < 0.001 uncorrected; Figure b. For two participants, the threshold of *p* < 0.001 resulted in too few voxels so the threshold was relaxed to *p* < 0.01.

For each participant, a general linear model with 20 factors (8 single-object conditions and 12 object-pair conditions) was applied to the fMRI data from the main experiment, and beta values were extracted from each stimulus block in each run and in each voxel of LO.

To equate the number of voxels across participants and to increase power, we selected 75 most reliable voxels in each ROI using reliability-based voxel selection^30^. This is based on the fact that, across the participants, the LO ROI ranged from 75 to 240 voxels before voxel selection. This method selects voxels whose response profiles are consistent across odd and even halves of the runs and works well when there are around 15 conditions. To implement this method, for each voxel, we calculated the split-half reliability by first averaging the runs within the odd and even halves and then correlating the resulting averaged responses for all conditions (20 in total) across the even and odd halves. We then selected the top 75 voxels with the highest correlations. The 75 voxels chosen had a moderate split-half reliability with an average of around *r* = 0.20 across the voxels and across the participants. We also analyze the data including all the voxels in LO and found virtually identical results.

#### CNN details

We tested 5 CNNs in our analyses (see Table 1). We examined both shallower networks, including Alexnet^25^ and VGG-19^26^, and deeper networks, including Googlenet^27^ and Resnet-50^28^. We also included a recurrent network, Cornet-S, that has been shown to capture the recurrent processing in macaque IT cortex with a shallower structure and argued to be one of the current best models of the primate ventral visual system^18,32^. All the CNNs used were pretrained with ImageNet images. As an additional test, because CNNs trained on ImageNet images are biased toward texture rather than shape processing, we evaluated 3 versions of Resnet-50 trained with stylized ImageNet images that emphasizes shape processing: Resnet-50 trained on Stylized ImageNet alone (RN50-SIN), Resnet-50 trained on both Original and Stylized ImageNet (RN50-SININ), and Resnet-50 trained on both Original and Stylized ImageNet, with fine-tuning on ImageNet (RN50-SININ-IN)^33^.

**Table 1:** CNNs and layers examined.

CNN name	Depth/blocks	Layers	N of total layers sampled	Sampled layer names and locations (indicated in the parenthesis)
Alexnet	8	25	3	**‘fc6’ (17)**, **‘fc7’ (20)**, ‘fc8’ (23)
Cornet-S	4	42	3	**‘IT_output’—IT (38)**, ‘decoder_avgpool’—avgpool (39), ‘decoder_output’—output (42)
Googlenet	22	144	3	**‘pool4-3 × 3_s2’—pool4(111)**, **‘pool5-7 × 7_s1’—pool5 (140)**, ‘loss3-classifier’—classifier (142)
Resnet-50	50	177	3	**‘activation_40_relu’—act40 (141)**, **‘avg_pool’—avgpool (174)**, ‘fc1000’ (175)
VGG-19	19	47	3	**‘fc6’ (39)**,**’fc7’ (42)**, ‘fc8’ (45)

All of the layers shown were examined in previous studies and are representative of the higher levels visual processing hierarchy^14,19,42^. Here, for each CNN, following Jacob et al.^29^, we only examined normalization in the higher-level layers, as only these layers show a large portion of units that respond to objects in both locations. Additionally, bold layers were those that showed best correspondence with LO as shown by Xu and Vaziri-Pashkam^14^.

In previous studies^14,19,40,42,46,47^, we sampled 6 to 8 pooling and fully connected (FC) layers representative of all stages of visual processing in these same CNNs and compared them with the human ventral visual processing areas. Following Jacob et al.^29^, who have previously examined averaging in CNN single units, here we included only the 3 highest layers we sampled previously, as only these layers contained a large portion of units that respond to objects at both locations (see Table 1 for the specific CNN layers sampled). We included pooling layers here because they typically mark the end of processing for a block of layers when information is pooled to be passed on to the next block of layers. When there were no obvious pooling layers present, the last layer of a block was chosen. It has been shown previously that such a sampling procedure captures the evolution of the representation trajectory fairly well, if not fully, as adjacent layers exhibit identical or very similar representations^47^. In each CNN, the layer(s) showing stronger correlation with LO in their representational structure for real-world objects than other layers tested in the same CNN^14^ were marked with bold font in all the results figures. For a given CNN layer, we extracted CNN unit responses to the 20 possible image conditions that the human participants saw in the scanner. These images all showed objects within a white rectangle on a gray background. As human participants could ignore the gray background and focus on the objects only but CNNs could not do so, we also extracted CNN unit responses to the same images on white backgrounds to assess the effect of background color on response averaging. Cornet-S and the three Stylized versions of Resnet-50 were implemented in Python. All other CNNs were implemented in Matlab. The output from all CNNs was analyzed and compared with brain responses using Python and R.

### Evaluating the unit response to single and paired objects

We followed the method of Zoccolan et al.^1^ and Jacob et al.^29^ to assess unit response to single and paired objects in single fMRI voxels in human LO and in CNN units (Fig. 1c). In LO, for each participant, we first averaged the beta weights across all the runs for each condition in each voxel. We then extracted paired and single object responses from each of the 12 object pairs and averaged across all the pairs to generate an averaged paired and single object responses for that voxel. Finally, we included all the LO voxels for a given participant and extracted the slope of the linear regression between a voxel’s response to a pair of objects and its summed response to the corresponding single objects shown in isolation. The resulting slope was then averaged across the participants. The average slope should be 0.5 if the single voxel response to an object pair is equivalent to the average response of the corresponding objects shown alone. In a previous single-cell analysis of monkey IT, a slope of 0.55 was reported^1^. We thus compared our slope results to both 0.5 and 0.55 as baselines. For each CNN layer examined, we only included in the regression analysis units that were responsive to objects at both locations; in other words, units that showed a non-zero variance across the four single object conditions at each of the two locations. We then compared the slopes of the linear regressions of the CNN layers exacted using the same procedure as outlined above with that of human LO.

### Evaluating the pattern response to single and paired objects

We followed the method of Jeong and Xu^7^ to assess pattern response to single and paired objects in fMRI response patterns in human LO and in CNN unit response patterns (Fig. 1d). In LO, to extract voxel response patterns, following established practice^48^, for each participant, we first z-normalized the beta values across all voxels for each condition in each run to remove amplitude differences between conditions and runs. We then divided the data into odd and even runs and averaged the runs within each half. We performed two correlations: correlating the voxel response pattern of the same object pair between odd and even runs (actual pair) and correlating the voxel response of an object pair and the average of its constituent objects shown alone between odd and even runs (averaged pair). To account for the fMRI measurement noise, we derived a noise-corrected correlation by dividing average pair correlation with actual pair correlation. If the noise-corrected correlation is no different from 1, it would indicate that the representation of an object pair is equivalent to the average representation of its constituent objects shown in isolation. This was done for each object pair and the results were averaged across all the object pairs for each participant. For CNNs, because there was no noise in the data, we simply calculated the response pattern correlation of an object pair and the average of its constituent objects. Similar to the CNN unit response analysis, we only included units that were responsive to objects at both locations. We then directly compared whether the correlations from the CNN layers were significantly different from that of the human LO.

To examine whether mechanisms other than a simple average or sum can predict the representation of an object pair, we additionally tested in both human LO and CNNs whether or not patterns generated by a weighted average model would better predict that of the actual object pair. To do so, we systematically varied the contribution of the two isolated objects’ patterns from 0 to 100%, with 10% increments in between. The resulting weighted average was then evaluated in the same way as before.

### Evaluating the relationship between unit response and pattern response

Here we examined whether fMRI voxels/CNN units with better averaging in response amplitude would exhibit better averaging in response pattern. To do so, we extracted the slope of the linear regression between an fMRI voxel/unit’s response to a pair of objects and its summed response to the corresponding single objects shown in isolation as before. We then sorted the voxels/CNN units into two groups based on whether their slopes were close to 0.5, defined as having a slope between 0.45 and 0.55 (referred to as the *near* condition), or whether they were far from 0.5, defined as having a slope less than 0.45 or greater than 0.55 (referred to as the *far* condition). Finally, we evaluated averaging in response patterns for the near and far voxels/units separately, and tested whether the near ones showed better pattern averaging than the far ones. We also noted the percentage of voxels/units that were either near or far.

#### Experimental design and statistical analyses

Ten human participants took part in the experiment. The factors described in the previous methods sections were evaluated at the group level using t-tests. One-tailed t tests were performed when the comparison in one direction was meaningful. We corrected for multiple comparisons in all post-hoc analyses using the Benjamini–Hochberg method^34^. For the fMRI analyses of LO, we corrected for two comparisons with a baseline level of normalization (0.5 or 0.55 for the unit response analysis and 1 for the population response analysis), accounting for including either all of the voxels or the top 75 most reliable voxels. For both the CNN unit and population analyses, for each CNN, we corrected for six comparisons with LO (three layers each and two background colors). When examining the relationship between CNN unit and population analyses, for each CNN, and for each background color, we corrected for nine comparisons (three layers and three groups of units included, i.e., near, far, and all units). We also calculated effect size using Cohen’s D^49,50^. All of the above statistical tests were conducted using R^51^.

## Data Availability

Data supporting the findings of this study are available at https://osf.io/nkdgf/.
